# Viral intra-host evolution in immunocompetent children contributes to human norovirus diversification at the global scale

**DOI:** 10.1080/22221751.2021.1967706

**Published:** 2021-09-02

**Authors:** Kentaro Tohma, Mayuko Saito, Monica J. Pajuelo, Holger Mayta, Mirko Zimic, Cara J Lepore, Lauren A. Ford-Siltz, Robert H. Gilman, Gabriel I. Parra

**Affiliations:** a Division of Viral Products, CBER, FDA, Silver Spring, MD, USA; b Department of Virology, Tohoku University Graduate School of Medicine, Sendai, Japan; c Department of Cellular and Molecular Sciences, Faculty of Sciences, Universidad Peruana Cayetano Heredia, Lima, Peru; d Johns Hopkins University Bloomberg School of Public Health, Baltimore, MD, USA

**Keywords:** Calicivirus, norovirus, evolution, intra-host evolution, immunocompetent, gastrointestinal infection

## Abstract

Norovirus is a major cause of acute gastroenteritis. Human noroviruses present >30 different genotypes, with a single genotype (GII.4) predominating worldwide. Concurrent outbreaks of norovirus are often associated with the emergence of new viruses. While different hypotheses have been presented, the source of new mutations in noroviruses is still unknown. In this study, we applied high-resolution sequencing to determine the intra-host viral diversity presented by noroviruses during the acute and shedding phase of infection in children. Profiling viral intra-host diversification at nearly full genome level indicated that GII.4 viruses presented dynamic intra-host variation, while non-GII.4 viruses presented minimal variation throughout the infection. Notably, the intra-host genetic variation during the shedding phase recapitulates the genetic diversity observed at the global level, particularly those mapping at the VP1 antigenic sites. Thus the intra-host evolution in healthy children explains the source of norovirus mutations that results in diversification at the global scale.

## Introduction

Human norovirus is one of the major causes of acute gastroenteritis worldwide. Its genome is a single-stranded, positive-sense, polyadenylated RNA molecule organized into three open reading frames (ORFs). ORF1 encodes the non-structural proteins (NS1/2-7) that are required for virus replication, and ORF2 and ORF3 encode the major (VP1) and minor (VP2) capsid proteins, respectively. The viral genome is enclosed in an icosahedral particle primarily formed by an array of the VP1 [1]. Norovirus VP1 protein presents two structural domains: the shell (S) domain forms the scaffold of the capsid, and the protruding (P) domain is an arch-like structure that extends from the S domain. The P domain is further divided into a conserved P1 and a variable P2 sub-domain, which contains the major B-cell epitopes involved in viral neutralization [2–4]. The role of VP2 remains to be elucidated, but it has been suggested to be involved in particle stabilization [5].

Genetic differences within VP1 are used to classify noroviruses into at least 10 genogroups (GI–GX) and multiple genotypes within each genogroup [6]. Although humans can be infected by different genotypes, a single genotype (GII.4) is predominant worldwide [7,8]. GII.4 dominance has been related to the continuous accumulation of mutations at major antigenic sites on the VP1, which results in the emergence of antigenically distinct variants [9–11]. This evolving pattern has only been seen in GII.4 viruses, as the VP1 protein from non-GII.4 viruses presents limited diversification even after decades of continuous circulation [12]. In addition to the variation on the VP1, changes in the RNA-dependent RNA polymerase (RdRp) and other non-structural proteins have also been associated with the emergence of new predominant viruses [13,14]. Thus norovirus diversification could also be achieved by genome exchanges among different polymerase and capsid types by means of recombination at the ORF1/ORF2 boundaries [7,15]. Notably, the ORF1/ORF2 recombination is restricted to viruses with high sequence similarities [16].

Concurrent outbreaks of norovirus occurring globally are usually associated with the emergence of a new virus, either by means of recombination or by point mutations. Several hypotheses have been suggested to explain the source of these new noroviruses. Immunocompromised patients can be infected for months or years, which result in the presence of very diverse noroviruses [17–21]. Thus one hypothesis is that immunocompromised patients could act as reservoirs for noroviruses with new mutations that would be transmitted to the healthy population. Whether these individuals could be the major source of new viruses to the global population remains to be determined [19,21–23]. Because there are multiple instances of human norovirus detected in animal samples and binding to animal intestinal tissues, another hypothesis is the spill-over of noroviruses from animal reservoirs [24,25]. While epidemiological data suggest the potential of such inter-species transmissions, recent phylogenetic analyses indicated strong species-specific segregation of noroviruses [7,26] and that the direction of the transmission is skewed to human-to-animal [27]. Finally, recent findings of evolutionary intermediate viruses, i.e. viruses that branched between major phylogenetic genotype clusters, in archival clinical samples collected from different countries since the 1970s suggest it is likely that novel noroviruses emerge from susceptible healthy individuals [16].

Although norovirus disease is characterized by sudden onset of symptoms that typically last 2–3 days, the virus could persist for several weeks or months [28–31]. This provides an opportunity for the virus to replicate and generate mutations that could be transmitted from person-to-person [32]. Intra-host norovirus diversity has not been extensively investigated in immunocompetent individuals, with only few studies examining viral diversity in a limited number of samples [12,17,18,32,33]. In this study, we collected a series of samples from norovirus-positive healthy children and performed high-resolution genome sequencing to gain insights on the contribution of intra-host evolution to the plethora of mutations and variants detected on a global scale.

## Materials and methods

### Dataset

Samples were collected in two cohort studies conducted in Peru and retrospectively analysed in this study. A birth cohort study was conducted in Las Pampas de San Juan de Miraflores, a shantytown in southern Lima, during 2007–2011, as described previously [28]. Briefly, pregnant women and those with newborns younger than 3 months were randomly selected from a complete community census. Exclusion criteria were hospitalization for >1 month at birth, any congenital defect, twin birth and birth weight <1500 g. Field workers visited each household and collected stool samples weekly until the age of 2 years. Additional samples were collected when children presented gastroenteritis symptoms. Written informed consent for her infant was provided from each enrolled woman [28]. Another cohort study of children younger than 2 years old was conducted in Villa El Salvador, a peri-urban community in the southern part of Lima, Peru, during 2013–2014. Known immunocompromised children were excluded from this study. Daily gastrointestinal symptom surveillance was conducted for cohort children by field workers, and stool samples were collected at the time of the diarrhea symptoms reported, and every two weeks for the first 2 months post-onset of the symptoms. In addition, monthly sampling was conducted for the course of the whole study. This study was approved by the Ethics Committees of Asociación Benéfica PRISMA, Universidad Peruana Cayetano Heredia, Johns Hopkins University, and Tohoku University Graduate School of Medicine (IRB numbers: 042-13, 58378, 1628 and 2020-1-150, respectively). In both studies, viral RNA was extracted from the stool samples and tested by one-step real-time PCR for the detection of norovirus GI and GII [28]. Stool samples were stored at –80°C at Universidad Peruana Cayetano Heredia.

### Quantitative PCR assays

Stool samples were available from 19 shedding episodes of norovirus acute infection in 15 children under 2 years of age; 4 of them (episodes 1, 5, 15, and 18) were cases of re-infections with different genotypes occurring in three distinct children (Table S1). Stool samples were shipped to the US Food and Drug Administration and suspended in 10% PBS buffer. The 90 µl of fresh RNA was extracted from 50 µl of the 10% stool suspension using MagMax Viral RNA Isolation Kit (ThermoFisher Scientific, Sunnyvale, CA, USA). To determine the virus genome titer and its dynamics during the shedding phase, the extracted viral RNA genome was quantified by quantitative PCR (qPCR) as described previously [2,34]. Plasmids harboring the full-length genome from GI (Norwalk virus; M87661) and GII (MD145-12; AY032605) were used as PCR standards for genome titration. Both plasmids were kindly provided by Dr. Kim Green (NIAID, NIH). The mean genome titer of duplicate wells was calculated using Bio-Rad CFX Maestro version 4.0. This retrospective study was approved by FDA IRB # 16-069B.

### Full-length RT-PCR and next-generation sequencing

Nearly full-length high-resolution sequencing was performed as previously described [12]. Briefly, complementary DNA (cDNA) was generated from the extracted viral RNA genome using the Maxima Minus First Strand cDNA Synthesis Kit (ThermoFisher Scientific) and the poly-A primer. The full-length viral genome amplification (40 cycles) was done using the SequalPrep Long PCR Kit (Invitrogen) and primers GII 1-35, GI 1-36, and Tx30SXN [12,16]. The resulting full-length viral genome amplicons (∼7.5 kb) were run on agarose gels and extracted using Qiagen Gel Extraction Kit (Qiagen, California, USA). The gel-extracted amplicons were quantified using the Qubit dsDNA HS Assay Kit (ThermoFisher Scientific) and subjected to next-generation sequencing (NGS) using MiSeq system (Illumina, California, USA). The library for NGS was prepared using the Nextera XT DNA Library Prep Kit (Illumina), and the paired-end 2 × 250 bp sequence reads were obtained. Reads were quality-filtered (base quality score ≥ 20 and depth of coverage ≥ 10) and mapped against (i) a panel of reference norovirus genomes to screen the amplified genotypes and (ii) corresponding full- or nearly full-length reference genomes to reconstruct its consensus sequence using the HIVE platform [35]. The generated NGS reads obtained in this study were deposited in the Sequence Read Archive (BioProject accession number: PRJNA705706).

### Intra-host single nucleotide variant analyses

To determine the intra-host viral dynamics, the intra-host single nucleotide variants (iSNVs) within samples were quantified using HIVE platform [35]. As sequencing errors could arise from the reverse transcription (RT) and/or DNA amplification steps [36], we additionally quality-filtered using depth of coverage ≥ 100 and minimum frequency of iSNVs ≥10%. The minimum frequency was set to be 10% as a conservative cutoff based on the control experiments using norovirus full-length RNA transcript (GII.4 MD145-12 [37]) and DNA plasmids harboring GI.1 (Norwalk virus) and GII.4 (MD145-12) genomes. The iSNVs from individual samples were then mapped against corresponding consensus sequences and profiled based on the position on the genome, type of the mutations (nonsynonymous or synonymous mutations), temporal dynamics (*de novo*: newly generated; persisting: iSNVs observed in previous time point; and purged: iSNVs not detected in subsequent time point), and summarized using R v3.6.0. For quality control purposes, seven samples were repeated with the same process to confirm the reproducibility of the sequencing platform utilized. Statistical analyses were conducted using R and GraphPad Prism v7. The iSNV distribution on VP1 and the frequency of those shared among multiple episodes were subjected to permutation test with 100 replicates of simulation using R. In the simulation, same amount of iSNVs per episode was randomly sampled from VP1 under a null hypothesis, which assumed equal probability of mutation per site.

### Global sequence analyses

To associate the iSNV distribution and the viral diversification pattern detected on a global scale, archival VP1 sequence information from GII.4 (*n* = 1572), GII.6 (*n* = 160), and other non-GII.4 viruses (*n* = 552) deposited in the public database was collected as described previously [10,16]. Sequences from animals, environment, and immunocompromised patients were excluded from the analyses. The phylogenetic relationship among viruses sequenced in this study (intra-host viral population) and those from surveillance studies (global viral population) was inferred by maximum-likelihood phylogenetic trees as implemented in PhyML [38] using VP1 amino acid sequences. Genotypes were assigned using the Norovirus Typing Tool [39]. Variants of GII.4 viruses were assigned using the Norovirus Typing Tool [39] and/or amino acid differences on the complete VP1 protein with cutoff = 5% [12]. The frequency of the mutations, distribution of those on the genome, and timing/history of their appearance in the human population were compared against iSNV profiles detected in this study. The permutation test was performed to evaluate if the intra-host mutations were contributing to the global viral diversity; thus intra-host amino acid changes were simulated under a null hypothesis that assumed random mutations to occur at any positions on the VP1 protein. The frequency of random-generated iSNVs in global dataset was calculated and compared with those of observed iSNVs to assess the statistical significance of intra-host mutations on viral diversification at the global level. The *P* values were calculated with 100 replicates of simulation using R.

### Intra-host viral genome analyses in immunocompromised patients

To address the potential role of (lack of) host immunity on intra-host diversification, we analysed intra-host genetic diversity of GII.4 noroviruses detected in immunocompromised patients, which presented chronic infections for 4–20 months. The sequence information used was retrieved from publicly available databases collected in four different studies [18–21]. The phylogenetic and iSNV analyses similar to those conducted in immunocompetent children (see above) were performed using consensus or clonal viral sequences from immunocompromised patients.

## Results

**Figure 1.**
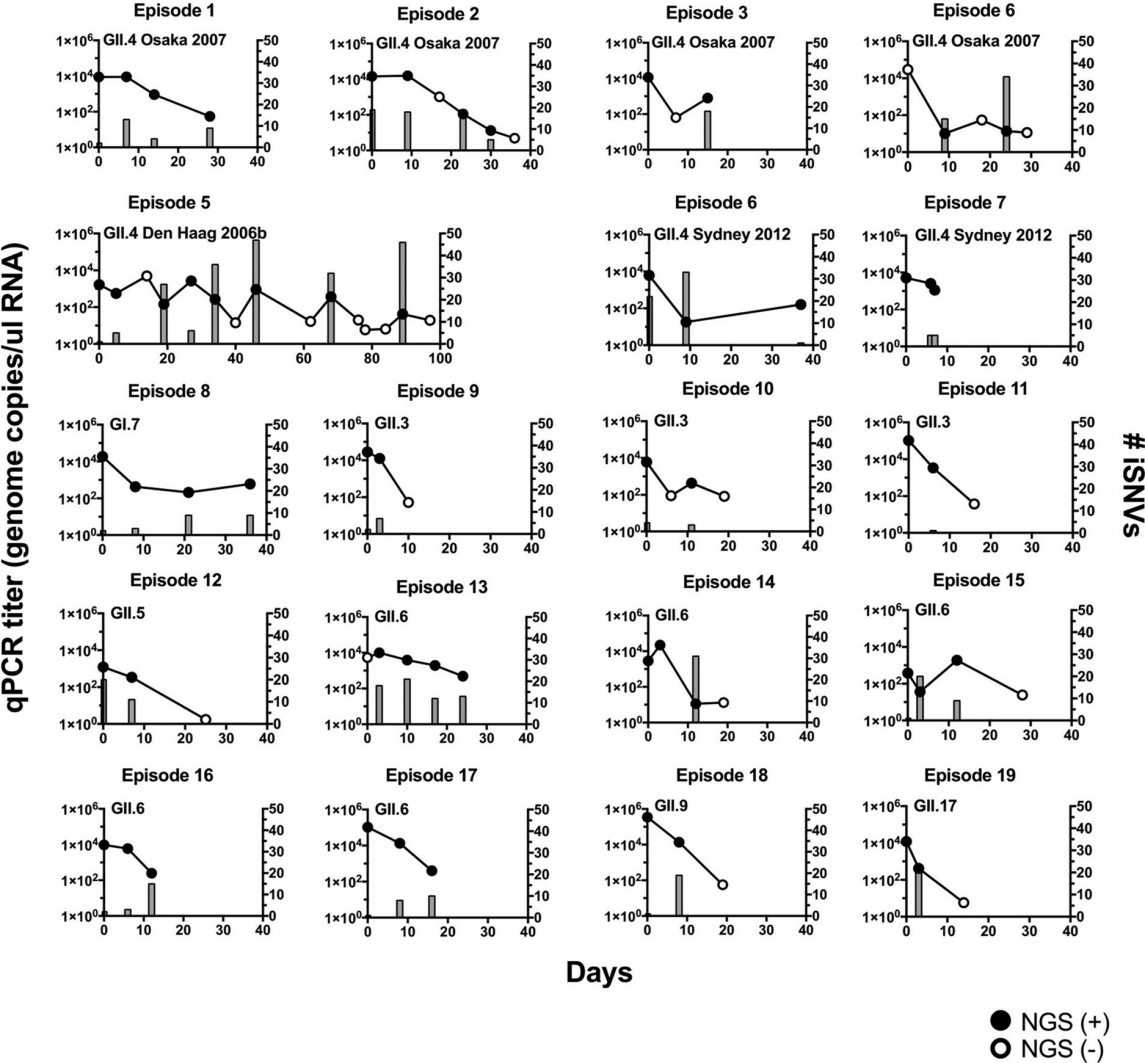
Norovirus genome copy numbers and virus intra-host single nucleotide variants (iSNVs) were detected in stool samples from infected children. The genome copy number (left *y*-axis, circles and lines; the mean of duplicate wells) and the number of iSNVs (right *y*-axis, bars) were tracked in individual infection cases (episodes). The filled circles indicate qPCR and genome sequencing positive samples, while empty circles indicate qPCR positive but genome sequencing negative samples. The corresponding genotype and variant information are indicated for each episode.

We analysed 78 stool samples from 15 norovirus-positive children, regardless of the symptoms, which enabled us to follow up the viral infection for 19 episodes. In this study, 19 episodes corresponded to infections with 7 different genotypes (GI.7, GII.3, GII.4, GII.5, GII.6, GII.9, GII.17) and 3 GII.4 variants (Osaka 2007, Den Haag 2006b, Sydney 2012; Table 1). Shedding of viruses was quantified using qPCR, and the length of infection varied from 8 to 98 days (average 26 days). Overall, the genome copies declined over time (Figure 1; line plot in left *y*-axis), with the exception of the GII.4 Den Haag 2006b infection that showed fluctuations in the number of genome copies during the infection.

**Table 1.** Norovirus infection episodes analysed in this study.

Episode	Child	Age (months)	Date of first norovirus positive	Duration of shedding (days)	Genotype [polymerase type]	Accession number^a^
1	PX164	5	2008-02-26	29	GII.4 Osaka [P4]	MW305524
2	PX011	11	2008-03-02	37	GII.4 Osaka [P4]	MW305538
3	PX212	4	2008-06-01	16	GII.4 Osaka [P4]	MW305545
4	PX044	14	2008-07-12	30	GII.4 Osaka [P4]	MW305521
5	PX212	5	2008-06-25	98	GII.4 Den Haag [P4]	MW305546
6	NV141X	5	2013-06-03	38	GII.4 Sydney [P31]	MW305535
7	NV010X	14	2014-01-25	8	GII.4 Sydney [P31]	MW305527
8	NV148X	15	2013-10-10	37	GI.7 [P7]	MW305488
9	PX061	17	2008-11-07	11	GII.3 [P21]	MW305513
10	PX067	23	2009-05-14	20	GII.3 [P21]	MW305541
11	NV077X	14	2013-09-17	17	GII.3 [P16]	MW305536
12	NV063X	17	2013-10-04	26	GII.5 [P22]	MW305533
13	PX164	2	2007-11-16	25	GII.6 [P7]	MW305523
14	PX029	16	2008-10-02	20	GII.6 [P7]	MW305540
15	PX212	8	2008-10-11	29	GII.6 [P7]	MW305547
16	PX177	11	2008-10-16	13	GII.6 [P7]	MW305544
17	NV042X	10	2013-09-16	17	GII.6 [P7]	MW305529
18	PX028	16	2008-09-22	9	GII.9 [P7]	MW305512
19	PX028	16	2008-09-05	12	GII.17 [P13]	MW305520

^a^ Genomes of viruses from the first qPCR/NGS positive sample obtained during the shedding episodes.

### Quality assessment of full-length NGS platform

Before proceeding to the stool samples, we performed a series of experiments to determine the robustness of our sequencing platform. First, sequencing error rate was evaluated using an RNA transcript and a plasmid that contains the full-length norovirus genomes. An overall highest frequency of intra-sample SNV was determined for multiple concentrations of full-length DNA template (5%) and of full-length RNA template (10%), and with this cutoff frequency, artificial intra-sample SNV was detected only from 0.025% of full-length sites (Fig. S1a). Second, we repeated the whole process of sample preparation and sequencing for three GII.4 and four GI.7 samples (Fig. S1b), and found that multiple iSNVs which presented a very high frequency (20–50% of the reads) were only detected in one of the duplicate runs. To investigate if those iSNVs were derived from actual viral population or sequencing errors, we calculated the ratio of nonsynonymous and synonymous mutations among iSNVs detected in either or both of the duplicate runs. Assuming that sequencing errors could arise randomly at any codon position, false-positive mutations could skew the distribution of mutations toward nonsynonymous changes. With the cutoff frequency = 10%, the ratio of mutations observed was well balanced and similar to that observed in both duplicate runs (Fig. S1b), suggesting minimal sequencing errors under this cutoff value, while also demonstrating high variability among aliquots and the difficulty of analysing the virus diversity in fecal samples [40]. Based on this initial data we used a conservative cutoff, ≥10% frequency, and a minimal of 100 reads per position for iSNVs calling. In the subsequent analyses, 58 out of the 78 norovirus-positive samples were successfully sequenced at nearly full-length level (≥7322 nt), with an average depth of coverage ranging from 835 to 60,292 (Table S1). The numbers of iSNVs in stool samples were quantified (Figure 1; bar plot in right *y*-axis), and neither the genome copy numbers in given samples, DNA concentration of full-length PCR amplicons, nor depth of sequence read coverage biased the number of iSNVs (*P* ≥ 0.07 in Pearson correlation test, Fig. S1c). Thus the iSNV profiles and temporal dynamics were compared among shedding episodes from different children with different norovirus genotypes.

### Differences in genetic robustness result in different patterns of intra-host evolution

**Figure 2.**
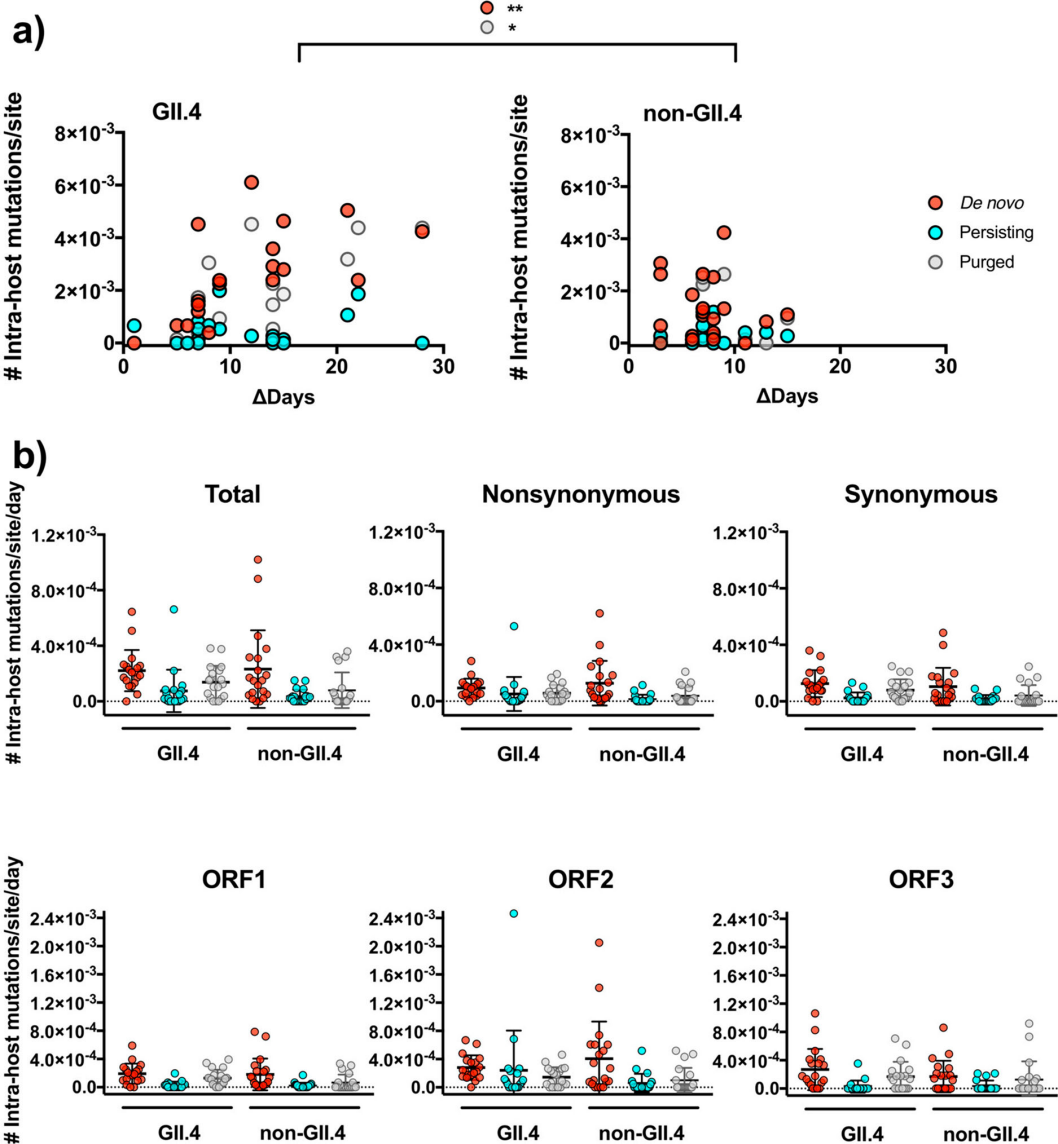
GII.4 noroviruses presented a higher number of mutational turn-over during the shedding phase of the infection. (a) The intra-host mutations were profiled into *de novo* (newly generated iSNVs), persisting (iSNVs observed in previous time point), or purged (iSNVs not detected) as compared to the mutational profile from the previous sample. The number of mutations was plotted on the *y*-axis with time intervals between corresponding samples indicated on the *x*-axis. *P < 0.05 and **P < 0.01 in one-way ANOVA and Sidak multiple comparisons test. (b) The dot plots summarize the number of intra-host *de novo*, persisting, and purged mutations by genotype, types of mutation, and ORFs. The lines and error bars indicate mean and standard deviation, respectively.

iSNVs were detected throughout the infection (Figure 1 and iSNV distribution shown in Fig. S2). To determine the evolutionary patterns detected at the intra-host level, iSNVs were profiled by the time of detection, position (ORFs), and type of mutations (synonymous or nonsynonymous), and were compared among different genotypes. Because major differences on the inter-host evolution were shown for norovirus GII.4 and non-GII.4 genotypes [12], we focused our analyses on these two groups. When plotting the iSNVs during the shedding phase, a drastic difference was noted between GII.4 and non-GII.4 viruses (Figure 2a). The iSNVs in individual episodes are summarized in Fig. S3, and profiles by mutational pattern and ORFs are summarized in Fig. S4. In both GII.4 and non-GII.4 viruses, several intra-host mutations persisted in the host for days and weeks; however, the GII.4 viruses presented a higher turnover of mutations over time, as shown by the higher number of *de novo* and purged mutations present in the viral population during the shedding phase as compared to non-GII.4 viruses (mean number of *de novo* mutations: 2.59 × 10^−3^ mutations/site in GII.4 and 1.32 × 10^−3^ mutations/site in non-GII.4 viruses, *P* = 0.0026; mean number of purged mutations: 1.72 × 10^−3^ mutations/site in GII.4 and 6.04 × 10^−4^ mutations/site in non-GII.4, *P* = 0.0102 in one-way ANOVA and Sidak multiple comparisons test; Figure 2a). While a higher number of *de novo* mutations was still confirmed for GII.4 viruses when including only shedding samples with up to 15 days apart (mean *de novo* mutations: 2.35 × 10^−3^ mutations/site in GII.4 and 1.32 × 10^−3^ mutations/site in non-GII.4 viruses, *P* = 0.0156 in Sidak multiple comparisons test), this trend was owing to the longer period of shedding observed in GII.4 virus infections (Figure 2a). Both viruses presented similar turnover rate (#intra-host mutations/site/day) in nonsynonymous and synonymous form, as well as at different ORFs (Figure 2b).

**Figure 3.**
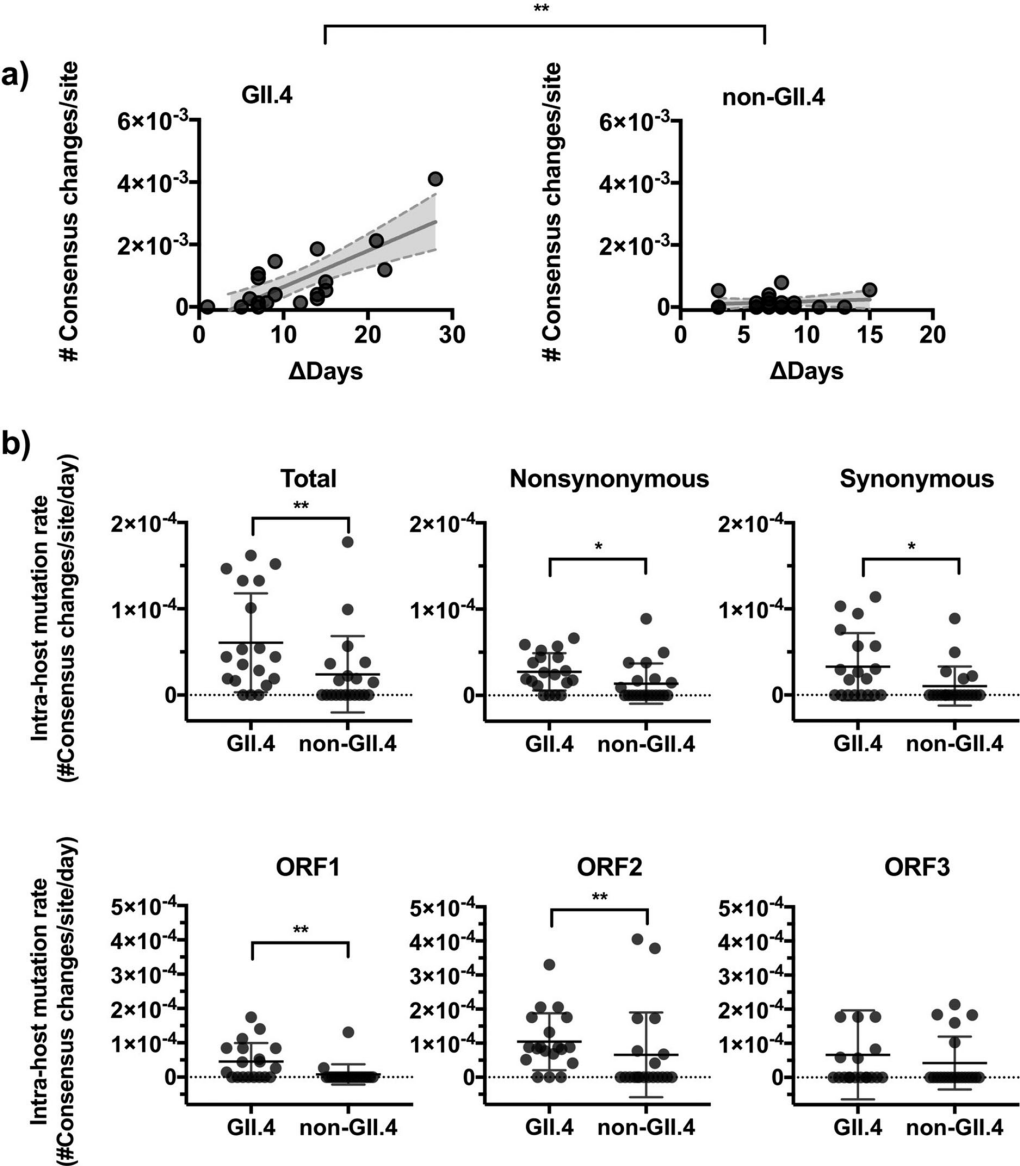
GII.4 noroviruses present an accumulation of nucleotide changes within the host. (a) The number of major nucleotide changes (frequency >50%) as compared to consensus genome sequence from the previous time point. The number of consensus nucleotide changes was plotted on the *y*-axis with time intervals between corresponding samples indicated on the *x*-axis. The lines in the graph indicate linear regression curves with 95% confidence intervals. (b) The dot plots summarize the number of intra-host consensus changes by genotype, types of mutation, and ORFs. The lines and error bars indicate mean and standard deviation, respectively. *P < 0.05 and **P < 0.01 in Mann–Whitney test.

Notably, considerable number of *de novo* iSNVs (143/569 in total) achieved consensus level (i.e. ≥50% intra-sample frequency). When tabulating the number of consensus mutations at each time point, GII.4 viruses achieved a higher turnover of dominant nucleotides throughout the infection (mean number of consensus mutations: 8.3 × 10^−4^ mutations/site in GII.4 and 1.53 × 10^−4^ mutations/site in non-GII.4, *P* = 0.0018 in Mann–Whitney test; Figure 3a). Further profiles by mutational pattern and ORFs showed that the differences were observed for both nonsynonymous and synonymous mutations, and at ORFs 1 and 2 (*P* < 0.05 in Mann–Whitney test; Figure 3b, individual patterns in Fig. S5). When tracking the diversification of the nucleotides within individual episodes, GII.4 viruses presented robust consensus changes, i.e. iSNVs were fixed (over 90% frequency) and/or purged (<10% frequency) afterward, while iSNV frequency of non-GII.4 viruses did not change drastically during the infections (Fig. S6).

### Intra-host diversification contributed to the antigenic evolution of GII.4 norovirus

**Figure 4.**
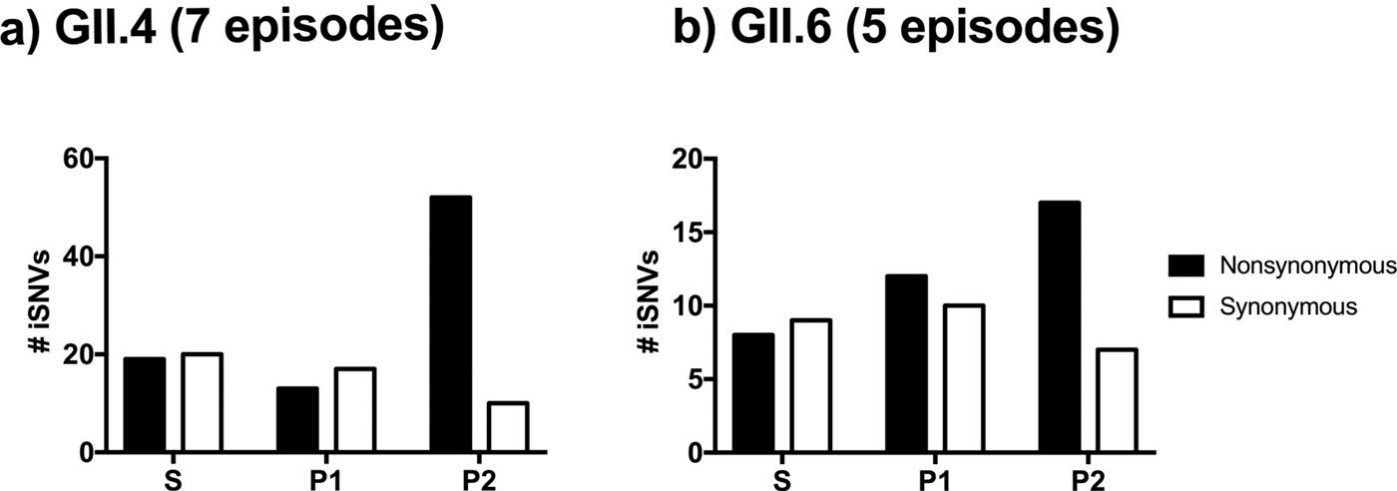
Nonsynonymous mutations are skewed on the P2 sub-domain on the GII.4 VP1 protein. The number of synonymous and nonsynonymous iSNVs in (a) GII.4 (7 episodes, 26 samples) and (b) GII.6 (5 episodes, 16 samples) viruses were summarized by the locations on the VP1 protein; S, P1, and P2 sub-domains.

The turnover of the predominant GII.4 variants on a global scale has been associated with the accumulation of amino acid changes on five major antigenic sites (A, C, D, E, and G) mapping on the VP1 capsid protein [9,10], resulting in antigenically distinct variants [11]. Thus the distribution of iSNVs from GII.4 viruses mapping to different regions of VP1 during shedding shows that most nonsynonymous mutations mapped on the P2 sub-domain (Figure 4a; *P* < 0.001 in Chi-square test), which harbors the major antigenic sites. The permutation test further supported significantly higher number of nonsynonymous intra-host mutations accumulated on the P2 sub-domain and antigenic sites while less mutations detected in the S and P1 sub-domains in GII.4 virus (Table S2). Interestingly, the nonsynonymous intra-host mutations on the antigenic sites were shared with different children (Table S2; *P* = 0.01 in permutation test), suggesting that antigenic sites are more dynamic than other residues at the intra-host level. On the contrary, iSNVs in GII.6, one of the most common non-GII.4 noroviruses, did not accumulate on the P2 sub-domain; rather, synonymous and nonsynonymous iSNVs were evenly distributed to the S, P1, and P2 sub-domains (Figure 4b; *P* = 0.277 in Chi-square test). The distribution of nonsynonymous iSNVs was not significantly different from those under a null assumption, random mutations at any sites on VP1 protein (*P *> 0.05 in permutation test), and not detected in parallel in different children (*P *> 0.05 in permutation test).

**Figure 5.**
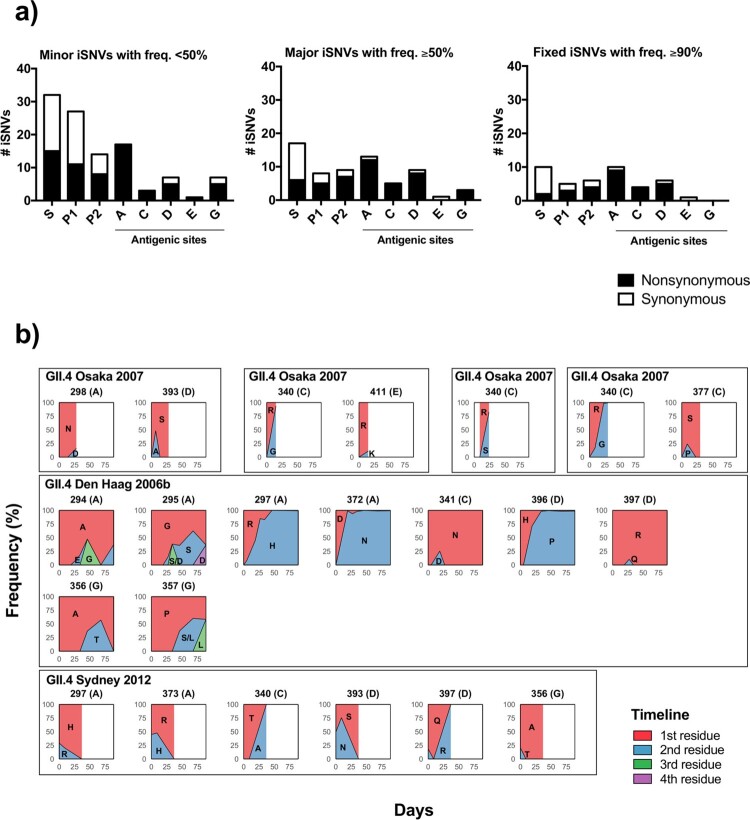
Major and minor intra-host mutations are differently distributed on the GII.4 VP1 protein. (a) The numbers of synonymous and nonsynonymous iSNVs were summarized by the locations on the VP1 protein: S, P1, P2 (excluding antigenic sites) sub-domains, and five major antigenic sites. Minor (frequency <50%), major (frequency ≥50%), and fixed (frequency ≥90%) iSNVs are summarized in different bar plots. (b) The intra-host amino acid mutational dynamics from residues mapping to major antigenic sites are shown for each nonsynonymous mutation. The area plots within the box indicate the amino acid changes detected in same child. The infected GII.4 variants, mutated positions, and corresponding antigenic sites are described for each area plot. The *x*-axis shows days since child was detected norovirus-positive. The *y*-axis shows the frequency of residues at each time point.

Further profiling by iSNV frequency in GII.4 virus showed that the surface-exposed P2 sub-domain and the five major antigenic sites presented both minor (frequency of <50%) and major (frequency of ≥50%) iSNVs and a higher number of nonsynonymous mutations (Figure 5a). On the other hand, most minor iSNVs mapped on the conserved S domain and P1 sub-domain, with more than half of them being synonymous mutations. The intra-host temporal dynamics of amino acid residues on the antigenic sites for the GII.4 infections are summarized in Figure 5b. Six out of the seven GII.4 shedding episodes reported iSNVs on the antigenic sites, with four of them presenting variation in at least two antigenic sites. In general, the sites that presented variability experienced two different amino acids. In ∼60% (13/22) of the changes, the predominant residue at the beginning of the infection persisted as predominant, and in the rest of the cases, *de novo* mutations dominated at the end of the shedding (Figure 5b). During the prolonged GII.4 Den Haag 2006b variant infection, three residues (294, 295, and 357) presented multiple amino acids co-existing throughout the shedding (Figure 5b).

### Intra-host diversification contributed to the diversity of norovirus on a global scale

**Figure 6.**
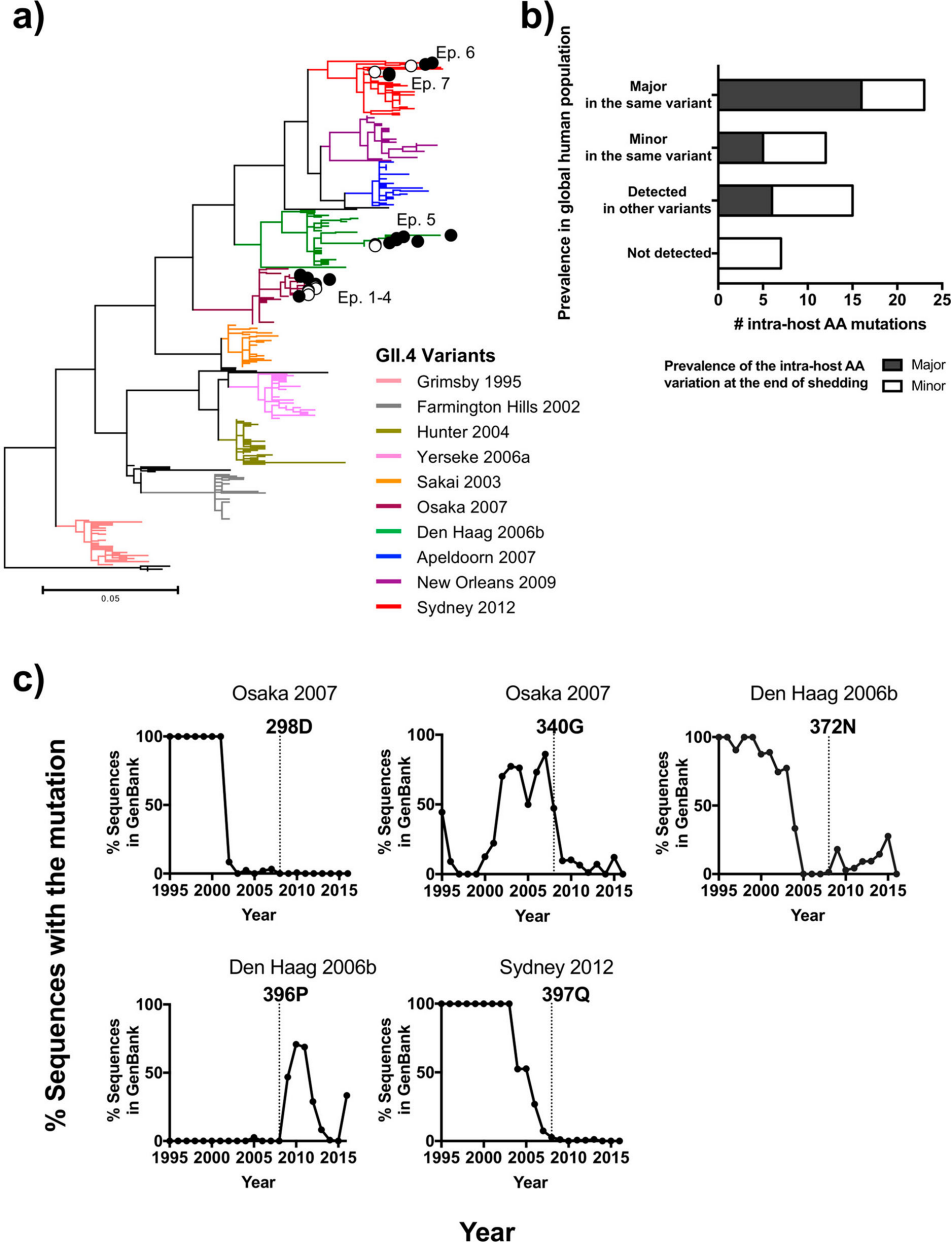
Intra-host mutations occurring in immunocompetent individuals are the source of GII.4 norovirus diversification on a global scale. (a) Maximum-likelihood phylogenetic tree of GII.4 noroviruses analysed in this study along with a global sequence dataset (*n* = 309 that were randomly selected from 1,572 complete VP1 sequences deposited in GenBank from viruses circulating during 1995–2016) indicated accumulation of amino acid changes within the host during the shedding phase; while being clustered together with viruses in a global population. Branches were colored by variants. Samples analysed in this study were denoted by circles with episode numbers; empty circles indicate viruses detected in initial samples and filled circles indicate those detected during the shedding phase of each episode. (b) Prevalence of the intra-host amino acid mutations on the P2 sub-domain in GII.4 noroviruses detected in the shedding phase was compared against observed mutations at the global level (*n* = 1,572 sequences). The bar plot shows the number of intra-host mutations in *x*-axis, colored by the prevalence of those at the end of the shedding; major intra-host mutations are indicated with black, and minor intra-host mutations with white. The *y*-axis indicates the prevalence of the same mutations detected using a global sequence database. From top to down: major amino acid residues detected in the same variant that infected the child; minor residues detected in the same variant infecting the child; not detected in the same variant but detected in other variants; and not detected in any GII.4 viruses. (c) The history of amino acid mutations mapping on the antigenic sites that were never detected in the same variant but dominated in the other variants was summarized in line graphs. The *x*-axis shows the year and the *y*-axis shows the ratio (%) of sequences with the specified residues in each year in the global dataset (black lines). The year of the corresponding intra-host mutation detected was indicated by a dot line in each graph.

Because GII.4 noroviruses exhibited continuous changes at the intra- and inter-host levels, we next investigated the fates of the intra-host viral divergence and nonsynonymous iSNVs mapping on the P2 sub-domain by analysing a global GII.4 sequence dataset from viruses circulating during 1995–2016 [10]. GII.4 noroviruses detected in the children all clustered within previously described GII.4 variants and presented accumulation of amino acid mutations during the shedding phase, while those were comparable to the ones detected at the global level (Figure 6a). The dominant amino acids at the end of the intra-host shedding were detected in the majority of circulating viruses, while minor amino acids within the host were detected as a minority in the same variant or detected in other variants (Figure 6b). A subset of amino acids that have been never detected at the global level were all minor and never dominated at the intra-host level, suggesting that those are most likely deleterious and eliminated during the transmission events [41]. A significant number of intra-host mutations were detected in the global dataset (*P* < 0.01 in permutation test, with a null model assuming random mutations on the P2 sub-domain), suggesting the contribution of intra-host evolution to the global viral divergence. Notably, intra-host mutations detected at a given variant could provide epidemic potential in previously circulated variants or in upcoming epidemic variants. For example, the intra-host mutation 298D detected in Osaka 2007 virus was detected in the majority of strains circulated back in the 1990s and early 2000s (Figure 6c). The intra-host mutation 396P detected in Den Haag 2006b virus was never detected at a global level but was a dominant residue (>80% of sequences) in the upcoming variant (New Orleans 2009; Figure 6c). On the contrary, intra-host diversity was limited in non-GII.4 noroviruses (Fig. S7), and the majority of the intra-host amino acid changes on the P2 sub-domain were minor and were not shown at high frequencies on a global scale (Fig. S8).

**Figure 7.**
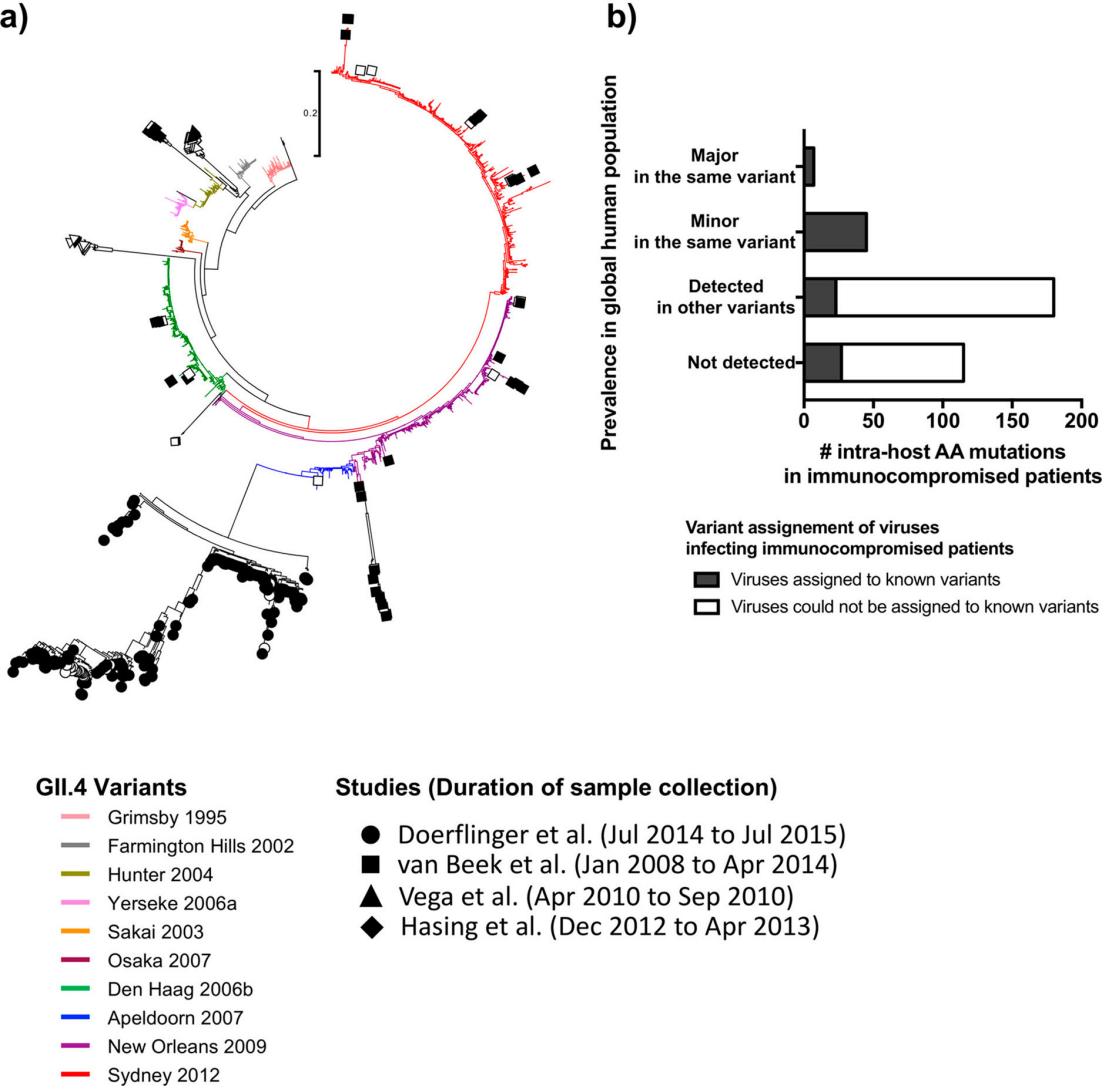
Intra-host mutations occurring in immunocompromised patients are not the source of GII.4 norovirus diversification on a global scale. (a) Maximum-likelihood phylogenetic tree of GII.4 noroviruses detected in immunocompromised patients along with a global sequence dataset (*n* = 1572) indicated disparity between viral diversity in immunocompromised patients and in a global population. Branches were colored by variants. Viruses detected from immunocompromised patients were marked based on the study [18–21]; empty symbols indicate viruses detected in initial samples and filled symbols indicate those detected during the chronic infection phase. (b) Prevalence of the intra-host amino acid mutations on the P2 sub-domain in GII.4 noroviruses detected from immunocompromised patients was compared against observed mutations in the global non-immunocompromised population. The bar plot shows the number of intra-host mutations in *x*-axis, and the *y*-axis indicates the prevalence of the same mutations detected using a global sequence database. From top to down: major amino acid residues detected in the same variant that infected the immunocompromised patients; minor residues detected in the same variant infecting the immunocompromised patients; not detected in the same variant but detected in other variants; and not detected in any GII.4 viruses in the global dataset. The bar plot was colored by the variant assignment of viruses infecting immunocompromised patients; viruses assigned to known GII.4 variants were indicated with black, and those that were genetically diverged and could not be assigned to known variants were indicated with white.

Notably, intra-host genetic diversity in immunocompromised patients was distinct from those in immunocompetent individuals and global population (Figure 7). Some of the viruses detected early in the infection were situated on the phylogenetic branches of the global population; however, intra-host diversification within the immunocompromised patients leads to genetically distinct viruses that do not clustered with any known variant. None of these viruses were detected afterwards to cause outbreaks in the immunocompetent population (Figure 7a). The majority of intra-host amino acid changes on the P2 sub-domain within the immunocompromised patients were not detected at a global level (Figure 7b), indicating that viruses generated within those patients are not spreading widely to the human population.

## Discussion

Norovirus is a fast-evolving pathogen and large outbreaks have been associated with the emergence of noroviruses with novel mutations, e.g. viruses with distinct antigenic differences from previously circulated strains [9–11], or those with different RdRp that could alter the transmissibility [13,42]. Because it was shown that intra-host variation in immunocompromised patients is higher than those from healthy individuals [17], it has been suggested that those patients could act as a reservoir for new norovirus that could spread worldwide [22]. Importantly, the norovirus variation detected in immunocompromised patients was not detected in the global dataset (Figure 7) [19,21], nor does it explain the differential pattern of evolution (namely, “static” and “evolving”) shown by non-GII.4 and GII.4 virus on a global scale [12]. A subset of mutations was only detected in immunocompromised patients and transmission of viruses with those residues was very limited in a global population. This could be due to changes in the immune pressure that restricts the infection of these viruses in immunocompetent individuals. Here, we provide substantial evidence that the intra-host diversification of noroviruses during the shedding phase of the infection in immunocompetent individuals recapitulates the pattern of evolution and amino acid diversification detected at the global level; thus GII.4 virus presents continuous accumulation of mutations, while non-GII.4 virus presents limited diversification at intra-host and global levels [12,16].

The intra-host viral dynamics presented different iSNVs profiles when comparing GII.4 and non-GII.4 viruses. Thus GII.4 norovirus presented a higher number of *de novo* and purged intra-host mutations during the shedding period when compared to non-GII.4 viruses. Although the differences were suggested to be derived from the duration of shedding, the dynamic turn-over could have major implications on the characteristics of viruses in the fitness landscape within the host. Indeed, GII.4 viruses successfully achieved a turnover of dominant nucleotides within the immunocompetent host, while non-GII.4 viruses presented minor variations that persisted throughout the course of the infection. All these findings further support that GII.4 noroviruses could explore a greater sequence diversity as compared to non-GII.4 noroviruses, and they are consistent with the inter-host data collected using norovirus sequences detected worldwide [12]. The mechanisms behind the greater genetic robustness observed on the VP1-coding sequences from GII.4 norovirus merit further investigation [43].

Correlation between intra- and inter-host viral divergence is well documented for other viruses [44–46]. The intra-host viral population is highly dynamic in influenza A viruses [44], which are constantly evolving in the human population [47]. In contrast, lower intra-host diversity was reported for influenza B viruses [45], which overall presented less diversity at the population level [48,49]. This data is not restricted to RNA viruses, as it was shown that canine parvovirus, a single-stranded DNA virus, presented limited intra-host diversity that is associated with the low mutation rate of this virus at the inter-host level [46]. The dynamic iSNV turnover in noroviruses seemed to contribute to the diversity detected at the global level. Thus GII.4 viruses presented a higher number of nonsynonymous intra-host changes mapped on the P2 sub-domain from the capsid protein VP1, supporting the notion that changes in multiple antigenic sites occur in the evolution of GII.4 noroviruses [11,50]. In addition, intra-host changes on the antigenic sites were detected in parallel in different children more than expected by chance, suggesting their contribution to the global divergence of this virus. Particularly, the highest number of nonsynonymous iSNVs was detected on the antigenic site A, capturing the amino acid diversity of antigenic site A at the global level [10]. The S domain and P1 sub-domain did not tolerate large intra-host nonsynonymous variation, which reflected the conserved nature of these domains at the global level. In contrast, non-GII.4 viruses presented evenly distributed synonymous and nonsynonymous iSNVs on the entire VP1 protein. Many of those were minor mutations within the host, and not detected at the global scale. This could be due to deleterious effects on the function of the proteins, selection under host immune pressure, and/or reduced transmissibility due to the low frequency during the infections. Thus GII.4 noroviruses could acquire new mutations and specific residues can predominate at the intra-host and inter-host levels. New mutations that arise at the intra-host level could spread to the global population; however, as shown for influenza viruses [51], different fitness is expected in the background of different viruses as residues that dominated in one variant were subdominant in other variants. On the other hand, the “static” nature of non-GII.4 viruses at global scale could be reflected by purifying selection acting at the intra-host and inter-host levels.

The limitation of our study includes artificial errors in iSNV calling, variability among aliquots in fecal samples, and relatively low genome titer particularly at the end of viral shedding that provided difficulty in amplifying a full-length genome and higher errors in iSNV calling. With such limitations, we processed iSNVs with conserved cutoff filters to have better specificity; thus no significant confounding bias was observed between the number of iSNVs and qPCR titer, read depth, or concentration of the full-length PCR amplicons. Although all those assumptions could have provided less sensitivity in subsequent iSNV analyses, our data indicate that the intra-host viral divergence in immunocompetent children could be linked to the evolution of noroviruses on a global scale. GII.4 noroviruses presented a highly dynamic intra-host population, which contrasted with the narrow sequence space utilized by non-GII.4 viruses within the host. Although further studies, including analyses of norovirus intra-host diversification in wider space-time and age groups, analyses of the role of synonymous mutations or epistatic interactions on the immune selection of variants, experimental evaluation of fitness landscape of intra-host viral population, and investigation of inter-host transmission bottleneck, are required for a comprehensive picture of the origin of new noroviruses, our study could provide an alternative explanation to the source of mutations that are detected at the global level and that ultimately could lead to the emergence of new noroviruses with epidemic potential.

## Supplementary Material

supplementary_materials.zip
